# Leonurine Protects Bone Mesenchymal Stem Cells from Oxidative Stress by Activating Mitophagy through PI3K/Akt/mTOR Pathway

**DOI:** 10.3390/cells11111724

**Published:** 2022-05-24

**Authors:** Bingkun Zhao, Qian Peng, Dan Wang, Rong Zhou, Raorao Wang, Yizhun Zhu, Shengcai Qi

**Affiliations:** 1 Department of Stomatology, Shanghai Tenth People’s Hospital, Tongji University School of Medicine, Shanghai 200072, China; 1811372@tongji.edu.cn (B.Z.); pengqhyc@163.com (Q.P.); zr181801054@163.com (R.Z.); 2 Department of Plastic Surgery, Affiliated Hospital of Xuzhou Medical University, Xuzhou 221000, China; 3 Institute for Tissue Engineering and Regenerative Medicine, The Chinese University of Hong Kong, Hong Kong, China; wangmd@cuhk.edu.hk; 4 School of Biomedical Sciences, The Chinese University of Hong Kong, Hong Kong, China; 5 State Key Laboratory of Quality Research in Chinese Medicine, School of Pharmacy, Macau University of Science and Technology, Macau SAR 999078, China; yzzhu@must.edu.mo; 6 Department of Prothodontics, Shanghai Stomatological Hospital, Fudan University, Shanghai 200001, China; 7 Shanghai Key Laboratory of Craniomaxillofacial Development and Diseases, Fudan University, Shanghai 200001, China

**Keywords:** osteoporosis, leonurine, mitophagy

## Abstract

Osteoporosis bears an imbalance between bone formation and resorption, which is strongly related to oxidative stress. The function of leonurine on bone marrow-derived mesenchymal stem cells (BMSCs) under oxidative stress is still unclear. Therefore, this study was aimed at identifying the protective effect of leonurine on H_2_O_2_ stimulated rat BMSCs. We found that leonurine can alleviate cell apoptosis and promote the differentiation ability of rat BMSCs induced by oxidative stress at an appropriate concentration at 10 μM. Meanwhile, the intracellular ROS level and the level of the COX2 and NOX4 mRNA decreased after leonurine treatment in vitro. The ATP level and mitochondrial membrane potential were upregulated after leonurine treatment. The protein level of PINK1 and Parkin showed the same trend. The mitophage in rat BMSCs blocked by 3-MA was partially rescued by leonurine. Bioinformatics analysis and leonurine-protein coupling provides a strong direct combination between leonurine and the PI3K protein at the position of Asp841, Glu880, Val882. In conclusion, leonurine protects the proliferation and differentiation of BMSCs from oxidative stress by activating mitophagy, which depends on the PI3K/Akt/mTOR pathway. The results showed that leonurine may have potential usage in osteoporosis and bone defect repair in osteoporosis patients.

## 1. Introduction

Osteoporosis is characterized by loss of bone mass, skeletal fragility, and deterioration of structure, which is a systemic bone disease [1]. The elderly often suffer bone fractures from osteoporosis, including especially the spine, proximal femur (hip), and humerus fractures [2]. The prevalence of osteoporosis was reported to be 10% in 50-year-old people and 25% in over 80-year-old people in 2010 [3]. It has become an urgent health concern worldwide and contributes significantly to healthcare costs with the increasing aging population.

Today, increasing recognition points out that reactive oxygen species (ROS) are important to the regulation of cell function. Many diseases related to oxidative stress have been confirmed, including osteoporosis [4]. The moderate redox state could indicate the balance of bone metabolism that requires the coordinated action of different types of bone cells: osteoclasts, osteoblasts and osteocytes [5]. Aging, estrogen deficiency, radiation, chronic inflammation, and other factors would disrupt this balance, causing bone loss [6,7]. During the differentiation process, an overloaded ROS burden would alleviate cell differentiation capacity and induce osteoblasts dysfunction resulting in a reduction in bone formation [8,9,10]. Therefore, dysregulated ROS and/or antioxidant systems seem to contribute to bone loss. Antioxidants directly or by counteracting the action of oxidants contribute to osteoblast differentiation, cell mineralization, and the inhibition of osteoclast differentiation [11]. Given the significant role of antioxidants in osteoporosis, recent research focuses on their advantages in bone metabolism, and suggests that antioxidants could be useful in antiresorptive therapies for bone loss.

Oxidative stress is the result of uncontrolled ROS production and inadequate levels of antioxidants. Therefore, antioxidants were added to increase osteogenic differentiation and detoxify the microenvironment from oxidative stress by down-regulating ROS and accelerating differentiation [12,13]. Traditional Chinese medicine has been developed to prevent and treat osteoporosis for thousands of years. The use of these extracted active compounds showed fewer side effects and higher sustainability [14,15,16], all of which can be used as antioxidants. Leonurine is a natural chemical compound extracted from the traditional Chinese medicine leonuri. Its high effeciency in the antioxidative effect has drawn much attention, including for cardiovascular diseases, antherosclerosis, ischemic stroke [17,18,19], etc. Interestingly, leonurine can reduce bone loss by inhibiting osteoclast differentiation and promoting 3T3-L1 cell differentiation [20,21]. Our previous research confirmed the contribution of leonurine to osteoblast proliferation and differentiation [19]. However, the possible ability of leonurine in BMSCs to prevent osteoporosis through the antioxidative effect is still unclear.

Multiple studies have discovered that mitochondria deteriorate with age and produce an excess of ROS, resulting in a loss of respiratory activity and an accumulation of damage to mitochondrial DNA [mtDNA] [22]. In pathological states, mitochondria is a source of uncontrolled ROS formation that alters mitochondria by destroying the mitochondria membrane from the mitochondrial permeability transition pore [23,24]. On the other hand, mitochondrial dysfunction is one of the main targets of ROS [25]. These double nature characters would fuel a vicious cycle that once damaged by oxidative stress, mitochondria would produce ROS at a high rate and, in turn, damage more mitochondria to deteriorate the intracellular environment [26]. Therefore, maintaining a healthy mitochondrial function network is essential in the response to physiological adaptations and the conditions of oxidative stress. Mitophagy is an extremely important mechanism in the maintenance of mitochondrial quality that is responsible for the maintenance of mitochondrial homeostasis in disease [27]. Improving mitophagy is important for oxidation resistance and reducing ROS accumulation [28]. Impaired mitophagy has been shown to have a negative impact on osteoblast differentiation and mineralization [29,30]. Restoration of impared mitophagy would help alleviate bone loss, paving a new strategy for antiosteoporosis therapy [31]. It has been shown that mitochondrial homeostasis and the redox state were rescued by leonurine treatment in ischemic stroke [17]. Therefore, we hypothesize that leonurine facilitates proliferation and differentiation of oxidative stress-induced BMSCs depending on mitophagy.

In this study, the protection and molecular mechanism of leonurine in H_2_O_2_ stimulated rat BMSCs was evaluated by CCK-8, cell apoptosis, ALP, intracellular ROS level, real-time PCR and western blot. Meanwhile, the role of leonurine in inhibiting the progress of osteoporosis and promoting bone defect repair was studied in vivo.

## 2. Materials and Methods

### 2.1. Cell Preparation and Establishment of an Oxidative Stress Model In Vitro

BMSCs were extracted from the bone marrow of Sprague-Dawley (SD) rats at four weeks of age. Briefly, rats were immersed in 75% ethanol after sacrifice. The tibias and femurs were separated and the BMSCs were flushed from the bone marrow by syringes and cultured in Petri dishes. After 24 h of incubation, BMSCs were extracted from each plate. Cells were maintained in α-MEM (α-MEM, HyClone, Logan, UT, USA) with 10% foetal bovine serum (FBS, Gibco, New York, NY, USA) and 1% penicillin/streptomycin (PS, Gibco, New York, NY, USA) in cell incubator with 37 °C/5% CO_2_. A basic characterization of cells was performed using flow cytometry on BMSCs markers CD11b(−) (Abcam, Cambridge, UK), CD90(+) (Invitrogen, New York, NY, USA), CD45(−) (Milteny Biotechnology, Shanghai, China) and osteogenic and adipogenic differentiation assays. Cells were passaged when the confluency reached about 80% and the medium was replaced every three days. Cells within the passages between three to six days were used in the experiments.

To establish an oxidative stress model in vitro, 3 × 10^3^ BMSCs were seeded in 96 well plates per well with five replicates. The measurement was repeated overnight. Incubation with a cell counting kit with eight assays (CCK-8) was used to confirm whether the cell numbers were equal. The cells were then co-cultured with different concentrations of H_2_O_2_ from 0 to 500 μM for 4 h. The medium was replaced with 10 μL of CCK-8 solution dissolved in 200 μL of cell culture medium. It was then incubated after 2 h and measured under absorbance at 450 nm.

To inhibit mitophagy, cells were pretreated with 10 mM 3-methyladenine (3-MA) (MCE, New Jersey, NJ, USA) for 4 h, and to inhibit the PI3K pathway, cells were pretreated with 2 μM 740 Y-P (APExBIO, Houston, TX, USA) for 2 h.

### 2.2. Cell Vitality

#### 2.2.1. CCK-8 Assays

Cell viability was measured using CCK-8 (Dojindo, Kumamoto, Japan) according to the manufacturer’s protocol. After treatment, the medium was replaced with 10 μL of CCK-8 solution dissolved in 200 μL of cell culture medium. It was then incubated after 2 h and measured under absorbance at 450 nm.

#### 2.2.2. Double Live/Dead Staining

The cells were washed with phosphate buffered saline (PBS) (Sangon Biotechnology, Shanghai, China) and incubated with a LIVE/DEAD^®^ Viability/Cytotoxicity Assay Kit (Invitrogen, New York, NY, USA) for another 30 min at 37 °C. The results were then observed by fluorescence microscopy and merged with image J.

#### 2.2.3. Cell Apoptosis Analysis

Annexin V-FITC/PI double staining (BD Bioscience, New York, NY, USA) was applied to confirm the apoptotic effect of ROS and protection effect of leonurine according to the manufacturer’s protocol. After leonurine treatment, cells were washed with precold PBS after collection. They were then stained with 5 μL of Annexin V-FICT and 5 μM propidium iodide (PI) after resuspension with 1×binding buffer in the dark for 15 min at room temperature. The experimental data was detected and collected using a BD FACSCanto II flow cytometer (BD BioScience, New York, NY, USA).

#### 2.2.4. RNA Isolation and Quantitative Real-Time PCR (qRT-PCR) Analysis

Total RNA was isolated with the TRIzol extraction method (Invitrogen, New York, NY, USA) according to the manufacturer’s protocol, and the concentration was measured using the Nanodrop system (Thermo Fisher, Cambridge, UK). cDNA was reverse transcribed with a PrimeScript RT reagent Kit (TaKaRa, Dojindo, Kumamoto, Japan). The cDNA was amplified and recoreded with Hieff^TM^ qPCR SYBR^®^ Green Master Mix in an ABI 7500 Real-Time PCR System (Applied Biosystems, Foster City, CA, USA). The relative expression level was calculated by the 2^−ΔΔCt^ method.

#### 2.2.5. Extraction of Proteins and Western Blot Analysis

Total protein was isolated with an RIPA buffer containing protease inhibitor and phosphatase inhibitor after osteogenic induction for six days. The protein was separated in equal amounts and transferred into nitrocellulose membranes (Millipore Corporation, Massachusetts, MA, USA). Primary antibodies were incubated with membranes at 4 °C overnight and secondary antibody was incubated at room temperature for 1 h. The membranes were visualized using the Odyssey LI-CDR system. GAPDH (1:2000) and Caspase-3 were purchased from Cell Signaling Technology (CST, Boston, MA, USA). BAX(1:1000) was purchased from Proteintech (Proteintech, Chicago, IL, USA).

### 2.3. Cell Differentiation

An osteogenic medium containing 10% FBS, 1% penicillin/streptomycin, 10 nM dexamethasone (Sigma, St. Louis, MO, USA), 10 mM sodium-glycerophosphate (Sigma, St. Louis, MO, USA), and 50 g/mL ascorbic acid (Sigma, St. Louis, MO, USA) was used to induce osteogenic differentiation. After treatment, osteogenic induction medium was used in the following experiment for six days (ALP staining, real-time PCR and western blot) and 14 days (Alizarin red staining).

#### 2.3.1. Alkaline Phosphatase (ALP) and Alizarin Red Staining

ALP staining was carried out after six days of culture. Cells were stained with the ALP color development kit (Beyotime, Shanghai, China) according to the manufacturer’s protocols after being fixed with 4% paraformaldehyde for 10 min. After being stained for 15 min, cells were washed with PBS three times. Subsequent observation and image capture was carried out under phase-contrast microscopy. For Alizarin red staining, cells were harvested after 14 days, fixed in 4% paraformaldehyde for 10 min and stained with Alizarin red staining kits (Beyotime, Shanghai, China) for 60 min. Subsequent observation and image capture were carried out under phase-contrast microscopy.

#### 2.3.2. RNA and Protein Level Analysis

The cells were then cultured with medium containing different concentrations of leonurine for 20 h and subsequently cultured with osteogenic induction medium for six days. The osteogenic-related mRNA level of OCN, OPN, Runx2 and the osteogenic protein level OPN, Runx2 expression level were further confirmed. OCN sense: 5′-TGAGGACCCTCTCTCTGCTC-3′, antisence: 5′-GGGCTCCAAGTCCATTGTT-3′; OPN sense: 5′-ATCTGAGTCCTTCACTG-3′, antisense: 5′-GGGATACTGTTCATCAGAAA-3′; Runx2 sense: 5’-GCACCCAGCCCATAATAGA-3’, antisense: 5’-TTGGAGCAAGGAGAACCC-3′; GAPDH sense: 5′-CAGGGCTGCCTTCTCTTGT-3′, antisense: 5′-TCCCGTTGATGACCAGCTTC-3′. GAPDH (1:2000) was purchased from Cell Signaling Technology (CST, Boston, MA, USA). OPG (1:500) and Runx2 (1:500) were purchased from Abcam (Abcam, Cambridge, UK).

### 2.4. Intracellular ROS Measurements

#### 2.4.1. Intracellular ROS Determination

An ROS assay kit was purchased from Beyotime for the production of intracellular ROS. Cells were co-cultured in H_2_O_2_ with or without leonurine. The cells were stained in serum-free medium containing 10 M DCFHDA and cultured for 20 min at 37 °C. It was observed under microscopy, measured by flow cytometry.

#### 2.4.2. Analysis of Intracellular ROS related mRNA Level

Cyclooxygenase 2 (COX-2), NADPH oxidase 4(NOX4) was further confirmed. The primer sequences as follows: COX-2 sense: 5′- TGAGCATCTACGGTTTGCTG-3′, antisense: 5′- ATCATCAGACCAGGCACCA-3′; NOX4 sense: 5′- GCACAGTCCTGGCTTACCTTC′, antisense: 5′-AGCAGCAGCAGCATGTAGAAGAC-3′.

### 2.5. Mitophagy Accessibility

#### 2.5.1. Measurement of Mitochondrial Membrane Potential

The fluorescent probe, JC-1 (Merk, New Jersey, NJ, USA), was applied to measure the mitochondrial membrane potential according to the manufacturer’s protocol. 3 × 10^4^ cells were seeded in 24-well plates per well and followed by different treatment. The cells were then incubated with JC-1 staining solution (5 μg) for 30 min in the incubator. After that, cells were rinsed for twice with PBS before observation with a fluorescence microscope and analysis with BD FACSCanto II flow cytometer (BD BioScience, New York, NY, USA).

#### 2.5.2. Colocalization of Mitochondria and Lysosome

Mitochondrial and lysosome colocalization was measured by staining with mito-tracker green (C1048, Beyotime, Shanghai, China), lyso-tracker red (C1046, Beyotime, Shanghai, China) and DAPI (C1002, Beyotime, Shanghai, China) according to manufacturer’s instructions. Briefly, Cells were incubated in serum free medium with 150 nM mitotracker green (excitation at 436 nm), 50 nM lyso-tracker red (excitation at 538 nm) and 2 μg/mL DAPI (excitation at 360 nm). Fluorescent images were obtained with a microscope.

#### 2.5.3. Analysis of Protein Level

Mitophagy-related protein level PINK1, Parkin, P62 and LCA/B expression level of mitophagy activation was further confirmed. PINK1 (1:500), Parkin (1:500), P62 (1:1000) was purchased from Abcam (Abcam, Cambridge, UK). LC3 I/II (1:1000) were purchased from Cell Signaling Technology (CST, Boston, MA, USA).

### 2.6. Pathway Investigation

#### 2.6.1. Bibliometric Evaluation

We first analyzed leonurine related articles by advanced retrieval using the search term “Mitophagy” [Mesh] and “leonurine” [Supplementary Concept] (Pubmed, https://pubmed.ncbi.nlm.nih.gov/ at 2 April 2021) to include all current research about these two aspects. With this approach, we recorded and reviewed 2057 and 89 articles. All articles were analyzed using the VOS viewer version1.6.13 (VOSviewer, https://www.vosviewer.com/ at 12 April 2021, Centre of Science and Technology Studies of Leiden University, The Netherlands). The rank of relevance score at top 60% was taken into analysis. The PI3K (PDB ID:3LJ3) protein was chosen for the coupling studies. The protein structure was downloaded from PDB (Protein Data Bank, https://www.rcsb.org at 5 April 2021).

#### 2.6.2. PI3K/AKT/mTOR Pathway Analysis and Molecular Docking

PI3K/AKT/mTOR activation was confirmed in the next step. Antibodies against AKT (1:1000), p-AKT (1:1000) and p-mTOR (1:1000) were purchased from Cell Signaling Technology (CST, Boston, MA, USA). Antibodies against PI3K (1:1000) and p-PI3K (1:500) were purchased from Abcam (Abcam, Cambridge, MA, USA). The PI3K structure (PDB ID: 3LJ3) was download from RCSB Data Bank (Protein Data Bank, https://www.rcsb.org at 5 April 2021) and the leonurine structure was prepared by Autodock Tools 1.5.6. It shows ligand-binding flexibility with the binding pocket residues. The lowest energy conformations were used for analysis and a picture was generated by Pymol.

### 2.7. Experiments on Animals

#### 2.7.1. Animal Preparation

Ten-week-old Sprague Dawley rats were obtained from Shanghai SLAC Animal Laboratory and fed in Tongji University’s Department of Laboratory Animal Science. These experiments were approved by the local ethics committee according to the guidelines for the care and use of animals. All rats had been acclimatized to the new conditions for one week. Ovariectomy surgery and skull defect surgery (5 mm) were performed after anesthesia. The rats were then assigned to three different group: the control group (Sham surgery followed with PBS), the OVX (ovariectomized) group (ovariectomy followed with PBS), and the OVX+Leonurine group (ovariectomy followed with leonurine treatment). Vehicle or leonurine (15 mg/kg per day) was administered intraperitoneally for eight weeks. Rats were sacrificed after eight weeks and femurs and cranial bone were harvested for microCT and histological analysis.

#### 2.7.2. MicroCT and Histological Examination

The condition of the craniums and femurs was scanned with a micro-CT scanner (Skyscan 1172, Bruker microCT, Kontich, Belgium) with a layer thick 10 μm. Further analysis includes the new bone volume/total volume (BV/TV), trabecular number (Tb.N), trabecular thickness (Tb.T), trabecular separation (Tb.Sp) and structure.

The samples were embedded in paraffin after decalcification with 10% EDTA and sectioned at a thickness of 5 μm. H&E staining was performed to observe osteoporotic progress and bone healing.

### 2.8. Statistical Analysis

Statistical data, with a repeat of least three times, was analyzed using SPSS 20.0 (IBM, New York, NY, USA). Statistics of the two groups were analysed by unpaired Student’s *t*-test. More than three groups were analysed by one-way analysis of variance followed by the Bonferroni post-test. All data are represented by mean ± standard error (SEM). *p*-values < 0.05 were considered significant.

## 3. Results

### 3.1. Establishment of the Oxidative Stress Model In Vivo

**Figure 1:**
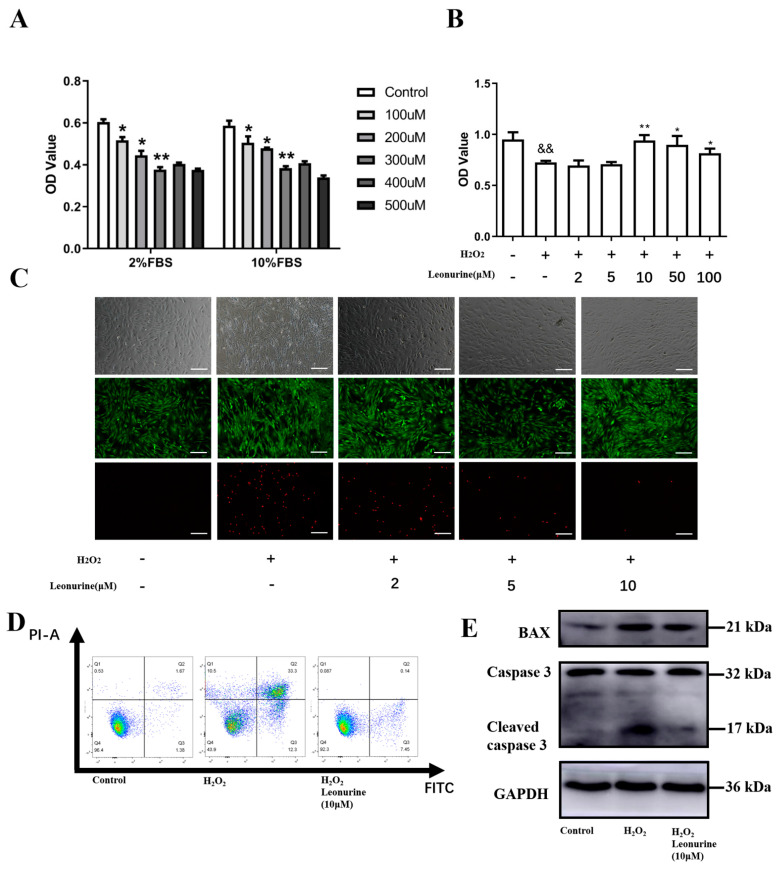
(**A**) CCK-8 essay for BMSCs co-cultured with different concentrations of H_2_O_2_. (**B**) Evaluation of leonurine protection at different concentrations in skeptical ROS burden. (**C**) Double live/dead staining (scale bar = 200 μM). (**D**) Distribution of apoptotic BMSCs observed under flow cytometry (FITC-Annexin V apoptotic detection assay). (**E**) Expression of apoptosis-related protein marker. Leonurine can help BMSCs survive from an overload ROS environment. (&& *p* < 0.01 vs. Control group. * *p* < 0.05, ** *p* < 0.01, vs. H_2_O_2_ group).

According to other reports, H_2_O_2_ was widely used to induce oxidative stress in vitro. We investigated different concentrations of H_2_O_2_ under both 2% FBS basic medium and 10% FBS normal culture medium. Cell vitality is shown to descend exactly to the platform in 300 μM for 4 h of treatment (Figure 1A). It laid the groundwork for the subsequent assessment of the leonurine function.

### 3.2. Leonurine Can Protect the Vitality of BMSCs from Oxidative Stress Damage

To access the protective effects of leonurine from ROS damage, we first tested its function on cell vitality. The effect of leonurine on BMSCs in vitro was investigated from 2 to 100 μM and it is shown that the protective effect is best when leonurine concentrations reach 10 μM (Figure 1B). We further carried out live/dead double staining and the trend is consistent with CCK-8 essays (Figure 1C). Cell apoptosis analysis showed an apparent decrease in the leonurine treatment group (Figure 1D). The apoptosis proteins like BAX and cleaved caspase 3 decreased in the leonurine group (Figure 1E).

### 3.3. Leonurine Can Protect the Differentiation Capacity of BMSCs from Oxidative Stress Damage

**Figure 2:**
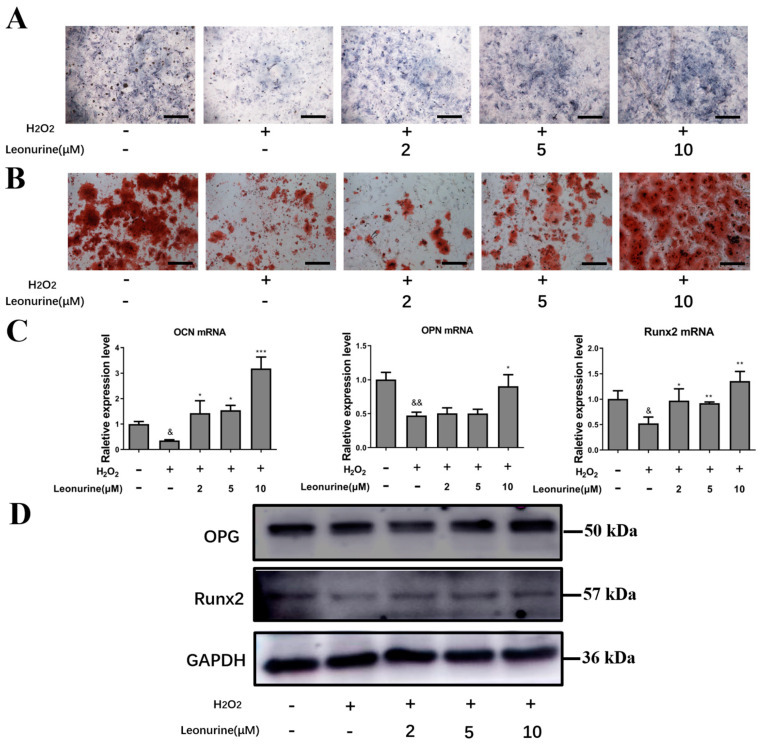
Effect of leonurine protection on BMSCs from ROS damage. (**A**) ALP staining of leonurine treated groups (0–10 μM) at day 6 (scale bar = 200 μM). (**B**) Aliza red staining of leonurine treated groups (0–10 μM) at day 14 (scale bar = 200 μM). (**C**) Osteogenic-related mRNA expression level. (**D**) Osteogenic-related protein expression level. (& *p* < 0.05, && *p* < 0.01 vs. control group. * *p* < 0.05, ** *p* < 0.01, *** *p* < 0.001 vs. H_2_O_2_ group).

To access the leonurine effect on the protection of osteogenic differentiation, ALP and Aliza red staining are carried out, accompanied by PCR and Western blot assay. From ALP and Aliza red staining, leonurine contributed to the protection of osteoblastic differentiation in a dose-dependent manner, which was the most apparent in the group treated with 10 μM leonurine (Figure 2A). Along the same lines, Alizarin red staining yielded comparable results, where a significant increase in mineralization was recorded for the 10 μM leonurine treated group (Figure 2B). Further evidence showed that the expression of osteogenesis-related markers increased at both mRNA levels (OCN, OPN, Runx2) and protein levels (OPG, Runx2) compared to the H_2_O_2_ group (Figure 2C,D).

### 3.4. Leonurine Can Alleviate Intracellular Oxidative Stress of BMSCs

**Figure 3:**
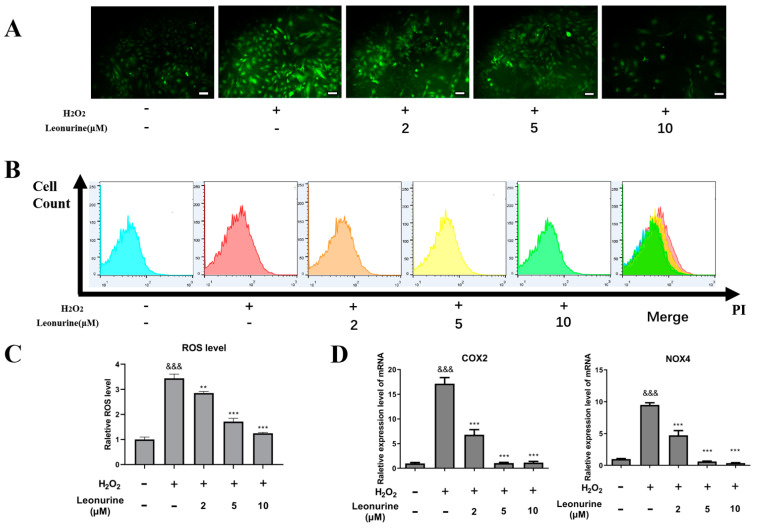
Effect of leonurine function on ameliorating ROS level. (**A**) Intracellular ROS measurement under microscopy (scale bar = 100 μM). (**B**) Intracellular ROS measurement by flow cytometry. (**C**) Quantitative analysis of flow cytometry results. (**D**) Intracellular ROS-related marker expression level. &&& *p* < 0.001 vs. control group ** *p* < 0.01, *** *p* < 0.001 vs. H_2_O_2_ group).

Considering the high oxidative content in patients with osteoporosis, we further tested the intracellular ROS level change. From intracellular ROS measurement assays, it showed that H_2_O_2_ would apparently increase intracellular ROS levels and leonurine can suppress ROS generation in a dose-dependent manner (Figure 3A). ROS analysis by flow cytometry indicated the similar results that leonurine can decrease ROS generation caused by H_2_O_2_ (Figure 3B,C). The reactive oxidative markers (NOX4,COX2) were used to test the intracellular ROS change in other respects, supporting the results of a decrease in the intracellular oxidative level in the leonurine treatment group compared with the H_2_O_2_ group (Figure 3D).

### 3.5. Leonurine Maintains Mitochondrial Quality Control by Activating Mitophagy

**Figure 4:**
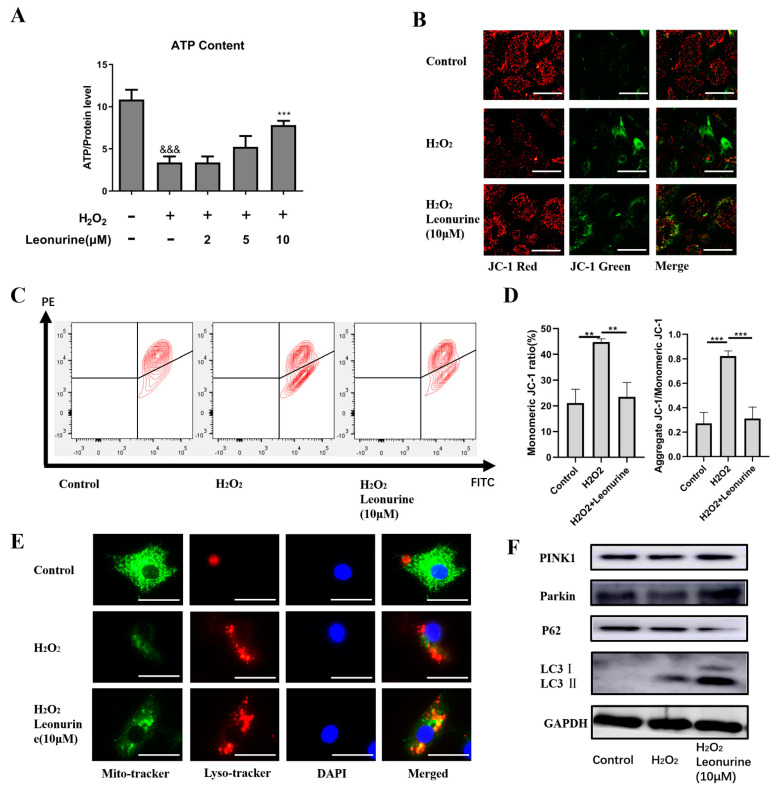
Leonurine activate mitophagy during ROS damage. (**A**) Intracellular ATP level (**B**) Measurement of mitochondrial membrane potential by microscopy (scale bar = 100 μM). (**C**) Measurement of mitochondrial membrane potential by flow cytometry. (**D**) Quantitative analysis of flow cytometry results. (**E**) Mitochondrial and lysosome colocalization analysis (scale bar = 50 μM). (**F**) Expression level of mitophagy-related proteins. (&&& *p* < 0.001 vs. control group. ** *p* < 0.01, *** *p* < 0.001 vs. H_2_O_2_ group).

Mitochondria, which is the first organelle to target ROS, is sensitive to intracellular ROS change. From the results, we found an obvious decrease in ATP level in the H_2_O_2_ group and an increase in ATP level in the leonurine group (Figure 4A). This result reflect that leonurine may act as a protective effect by maintaining mitochondrial quality control. The JC-1 assay indicated that BMSCs failed to maintain mitochondrial membrane potential, causing mitochondrial dysfunction (Figure 4B,C). In the leonurine treatment group, the mitochondrial membrane potential was maintained and mitochondrial dysfunction was suppressed (Figure 4B,C). According to the results, mitophagy was apparently activated in the leonurine group. We observed an increase in autophagy level (Red) both the H_2_O_2_ and leonurine group, but the leonurine group showed more fusion between mitochondrial (Green) and autophagosome (Red) (Figure 4E). Western blot experiments provided evidence for mitophagy activation at the protein level (PINK1, Parkin, P62, LC3 I/II) (Figure 4F).

### 3.6. Inhibition of Mitophagy Will Block Leonurine Function

**Figure 5:**
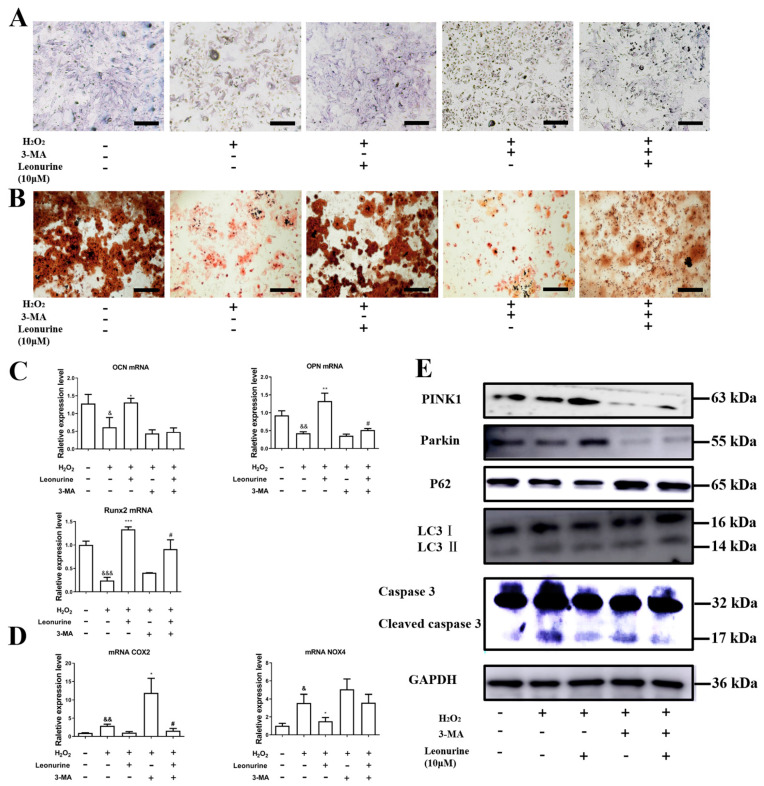
Effects of leonurine on mitophagy-inhibited BMSCs from ROS damage. (**A**) ALP staining on day six (scale bar = 200 μM). (**B**) Aliza red staining on day 14 (scale bar = 200 μM). (**C**) Osteogenic-related mRNA expression level. (**D**) Intracellular ROS-related marker expression level. (**E**) Related protein expression change (& *p* < 0.05, && *p* < 0.01, &&& *p* < 0.001 vs. control group. * *p* < 0.05, ** *p* < 0.01, *** *p* < 0.001 vs. H_2_O_2_ group. # *p* < 0.05 vs. 3-MA+H_2_O_2_ group).

Further experiments have identified the importance of mitophagy in the protection function of leonurine. From the results of the ALP and Aliza red staining, mitophagy inhibition would deteriorate the differentiation ability of BMSCs (Figure 5A,B). Leonurine partly rescued 3-MA-induced mitophagy inhibition and significantly declined the protection(Figure 5A,B). PCR analysis on osteogenic protein mRNA level consists of results of ALP and Aliza red staining, and leonruine, at least, partly rescued 3-MA-caused damage to the differentiation ability of BMSCs (Figure 5C). The next step in the analysis of the change in ROS level related to 3-MA indicated that 3-MA sharply increased the level of ROS-related markers(NOX4, COX2), and leonurine worked as an antagonist to 3-MA (Figure 5D). From the results of the western blot, 3-MA appeared to inhibit mitophagy, and leonurine partially reversed mitophagy inhibition (Figure 5E).

### 3.7. Analysis of the Leonurine-Implied Signaling Pathway

**Figure 6:**
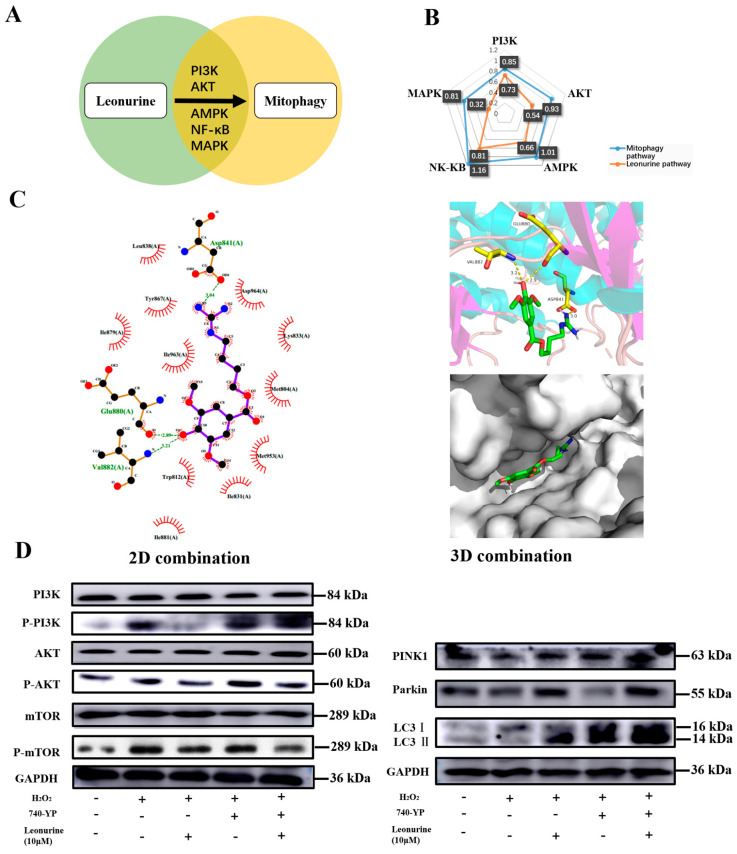
Leonurine can directly moderate PI3K-AKT-mTOR activation. (**A**) Research on the analysis of the leonurine and mitophagy pathway. (**B**) Relevance score on the analysis of the leonurine and mitophagy pathway. (**C**) 2D-molecular docking and 3D-molecular docking between leonurine and the PI3K protein. (**D**) The effect of leonurine on the PI3K-AKT-mTOR pathway and change in mitophagy.

We analyzed the research related to leonurine-related articles and found the five most related pathways (Figure 6A). Combined with the mitophagy relationship, it was shown that all the PI3K, AKT, AMPK, MAPK, NF-κB pathways have a strong relationship with mitophagy, and leonurine had a comparative high level of relation with the PI3K and NF-κB pathways (Figure 6B). We examined whether leonurine had a direct affinity for related pathways. Molecular coupling indicated that the chemical structure of leonurine can form a direction connection and dock nicely within the protein of PI3K (Figure 6C). The view of local interaction of protein residues was shown in a ribbon model. The 2D bonding model showed that some important hydrogen bonds were formed between the leonurine and amino acid residues (Glu880, Val882, Asp841) of PI3K with a high affinity of 3.04 kcal/mol, 2.89 kcal/mol, 3.21 kcal/mol. The PI3K-AKT-mTOR pathway had a high relationship with mitophagy. Therefore, western blot was used to identify leonurine function in the PI3K pathways (Figure 6D). Leonurine inhibits PI3K activation, including its downstream of AKT/mTOR, as well as the function of the PI3K activator (740-YP) in inhibiting mitophagy. These results supported leonurine moderate mitophagy through the PI3K/AKT/mTOR pathway.

### 3.8. Leonurine Improves Bone Healing under Osteoporosis Conditions In Vitro

**Figure 7:**
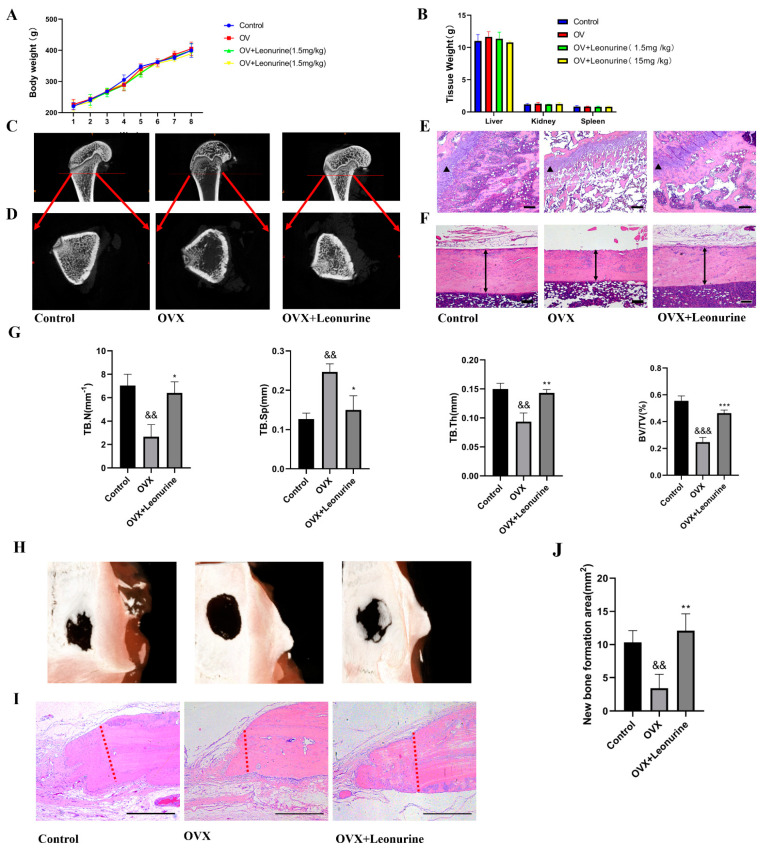
Leonurine ameliorates osteoporosis and contributes to osteogenesis in vivo. (**A**) Body weight. (**B**) Organic weight. (**C**) MicroCT of the femur on longitudinal section. (**D**) MicroCT of the femur on transection; (**E**) H&E staining of the femur on cancellous bone. (**F**) H&E staining of the femur in cortical bone. (**G**) MicroCT assessment evaluated for: Tb.N trabecular number; Tb.S trabecular spacing; Tb.Th trabecular thickness; BV/TV trabecular bone volume fraction; (**H**) Skull bone defect healing condition. (**I**) H&E staining of new bone formation. (**J**) Quantitative analysis of the new bone formation area. (&& *p* < 0.01, &&& *p* < 0.001 vs. Control group. * *p* < 0.05, ** *p* < 0.01, *** *p* < 0.001 vs. H_2_O_2_ group).

There is no apparent change in animal body weight of organic weight, and this indicated no apparent toxicity (Figure 7A,B). In the OVX rat model, leonurine inhibited bone loss, because we observed a higher bone density in cancellous bone (Figure 7C) and a thicker bone cortex (Figure 7D) in the leonurine group, which was consistent with the results of the H&E staining (Figure 7E,F). A microCT assessment including trabecular number (Tb.N), trabecular thickness (Tb.Th) and trabecular bone volume fraction (BV/TV) improved in the OVX+leonurine group compared with the OVX group, and trabecular spacing (Tb.S) apparently decreased, contrary to bone density (Figure 7G). These results showed that leonurine can apparently alleviate osteoporosis progression. To further confirm the bone healing ability, we next test bone formation based on a skull defect model to confirm the improvement in bone healing. The results showed that bone healing was inhibited by the osteoporosis condition, and leonurine can recover healing capacity (Figure 7H). From H&E staining, the OVX+leonurine group showed more new bone formation compared with the OVX group (Figure 7I), and the qualitative analysis showed a statistical difference (Figure 7J).

## 4. Discussion

Osteoporosis, a most common skeletal disordered disease, is becoming a major clinical and public health concern worldwide. The key feature of osteoporosis pathology is the contribution of oxidative stress to the onset and development of osteoporosis [32]. However, because of the involvement of high oxidative stress in osteoporosis pathogenesis and comorbidities, a novel therapeutic strategy arises where the treatment of different etiologic factors of osteoporosis can be possible with therapeutic targets [33]. In this study, we confirmed that leonurine can apparently decrease ROS level, protect BMSCs from apoptosis, and maintain the differentiation ability of BMSCs. Leonurine protection of BMSCs was achieved by inhibiting PI3K/AKT/mTOR to activate mitophagy. Meanwhile, the results showed that leonurine can alleviate osteoporosis and contribute to bone healing in the osteoporotic rat model in vivo.

For the establishment of the cell model, scientists used H_2_O_2_ [34], lipopolysaccharide(LPS) [35], and dexamethasone [36] to establish high oxidative stress. According to our results, H_2_O_2_ can apparently increase the intracellular ROS level of BMSCs, and many studies also use H_2_O_2_ as a suitable way to mimic the oxidative microenvironment in vitro [37]. Therefore, to investigate leonurine protection, we set a different concentration of H_2_O_2_ (100–500 μM) stimulating BMCSs in vitro, in which 300 μM H_2_O_2_ would reach the critical point concentration.

The mitochondria are the powerhouses of cells, acting as the source of ATP to maintain the cellular processes. In the pathological state, mitochondria is the prior damage target of ROS [38]. ROS can induce the loss of mitochondrial inner membrane permeability and an apparent drop in mitochondrial membrane potential (ΔΨm) [39]. Damaged mitochondrial membranes are also the major source of excessive ROS in cells, further causing deterioration of the intracellular microenvironment [10]. Due to the critical role of mitochondrial function, it has been confirmed that mitochondrial dysfunction has been associated with disease. Therefore, an increasing interest in the development of mitochondrial-directed therapeutics to correct or modify mitochondrial function was developed [40]. Leonurine, more specifically, restored mitochondrial function to attenuate cell damage [41]. Therefore, we also examined the high oxidative microenvironments surrounding BMSCs in osteoporosis patients and the high relation with mitochondrial quality control. Our hypothesis is confirmed by the fact that leonurine protects BMSCs from ROS damage by activating mitophagy. In microenvironments of oxidative stress, leonurine can alleviate the intracellular ROS level and protect BMSC proliferation and differentiation. From the results, we observed the mitochondrial fusion with the lysosome processing mitophagy procedure. Further research indicates that leonurine can at least partially reverse the mitophagy inhibition caused by 3-MA. Here we provided evidence to improve the osteogenic differentiation of BMSCs by mitophagy, which based on the importance and breakthrough in mitophagy involvement in scavenging damaged or burned-out mitochondria by preventing ROS overproduction [28]. From a previous report, mitophagy is becoming a new evident target in stem cell functions and a potential target in the progression of aging and diseases [42]. A recent study shows that mitophagy is directly essential to osteoblast mineralization [43]. This study revisits a 50-year-old conundrum and laid the solid foundation of osteoporosis therapy from mitophagy. As a result, evidence for treating osteoporosis by regulating mitophagy is growing [44]. Our research also proven that leonurine played the protection roles on BMSCs in vitro via regulating mitophagy in preventing ROS injury.

The mechanism of multiple pathways involves the regulation of mitophagy and is especially focused on the regulation of mTOR in bone metabolic disorder [45]. Leonurine has been reported to have a strong relationship with the PI3K pathway [21,46]. In this study, the results indicate a high relationship of leonurine with the PI3K-AKT-mTOR pathway. The results of the 2D and 3D docking studies also support that leonurine can regulate PI3K directly, causing downstream protein changes, and the results of the western blots indicated that the combination inhibits the phosphorylation of the PI3K/AKT/mTOR pathway. The PI3K pathway has been shown to be involved in the determination of cell endogenous ROS level and cell fate [47]. This pathway is thought to be important for cell survival and was thought to be a response to oxidative stress [48]. In particular, it is notable that our results are different. We observed that the inhibition of the PI3K-AKT-mTOR pathway by leonurine would improve the proliferation and differentiation ability of BMSCs. Indeed, it has been suggested that the PI3K pathway may be a double edged-switch depending on the different circumstances [49]. Therefore, more scientists are starting to notice this fact. The benefit of inhibition of the ROS-induced DNA damage activated PI3K pathway was observed in helicobacter pylori infection [50]. More research shows that inhibiting the PI3K/AKT/mTOR pathway has the same benefit in LPS-induced kidney injury, neurodegeneration, and osteoporosis [51,52,53]. According to our results, this study is focused on the downstream PI3K pathway in the mTOR protein. Long-term research on rapamycin, a well-known mTOR inhibition drug, for the treatment of osteoporosis in both experiments and clinical studies has added confidence in the clinical application of leonurine for the treatment of osteoporosis in the future [54]. Therefore, based on the previous reports and our findings, considering the different views on the PI3K pathway and the complexity downstream, further investigation of crosstalk between PI3K and mTOR should be clarified, which may be a key to explaining the conflicting questions about PI3K in different circumstances. Furthermore, it is worth clarifying further that mitophagy has different types of activation, including PINK1/Parkin and receptor-induced mitophagy such as NIX, BNIP3, FUNDC1 [55]. We only examined PINK1/Parkin, which has a close relationship with osteoporosis [44,56]. Due to the limited scope of this study, we were unable to include other types of mitophagy activation, and studies on receptor-induced mitophagy on osteoporosis are lacking, which needs to be further work in the future. What’s more, it is a pity that the cellular and dynamic bone histomophormometry were not quantified and we didn’t use micro-CT to evaluate the overall condition of cortical bone.

**Figure 8:**
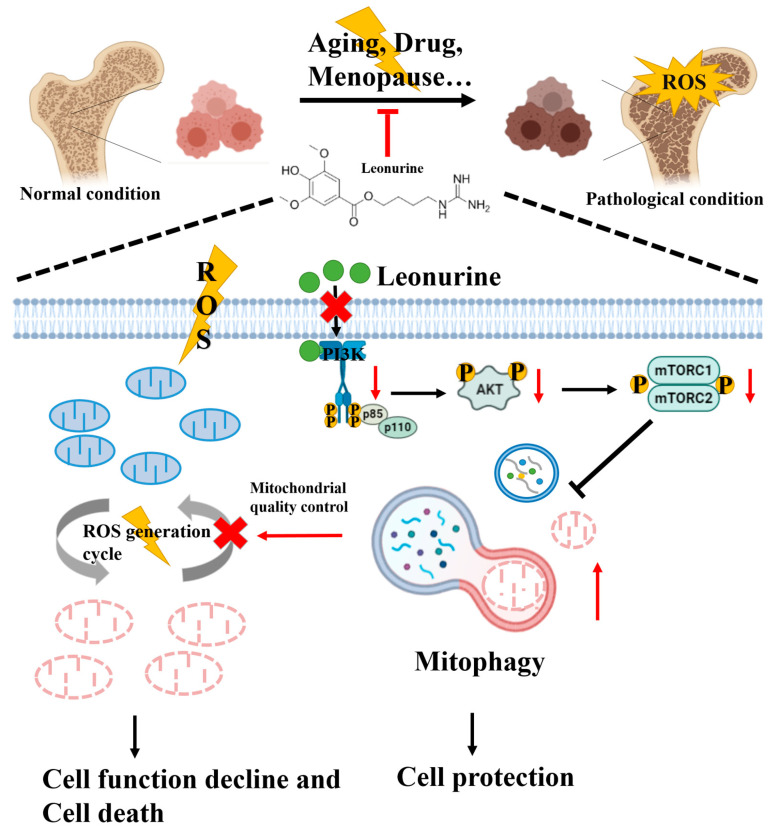
Mechanisms underlying our results. Leonurine ameliorates ROS damage to BMSCs by blocking ROS generation cycle by mitophagy to keep mitochondrial quality control. The mechanism probably involves the moderate activation of the PI3K-AKT-mTOR pathway. Therefore, leonurine can activate mitophagy to alleviate damage to BMSCs form ROS overload by the moderate PI3K-AKT-mTOR pathway.

In conclusion, our research revealed a new role for leonurine in the possible treatment of osteoporosis by activating BMSCs mitophagy. From the mechanism point of view, leonurine inhibits the PI3K-Akt-mTOR pathway to activate mitophagy, subsequently contributing to mitochondrial quality control by preventing the generation of intracellular ROS (Figure 8). These results strongly support that leonurine may be a candidate medicine for the development of new therapies for osteoporosis.

## Data Availability

The data can be further inquired directed to the corresponding authors. Data Prof. Shengcai Qi, Department of Stomatology, Shanghai Tenth People’s Hospital, Tongji University School of Medicine, 301 Yanchang Road, Shanghai 200072, China. E-mail: dentistqi@163.com; Prof. Raorao Wang, Department of Stomatology, Shanghai Tenth People’s Hospital, Tongji University School of Medicine, 301 Yanchang Road, Shanghai 200072, China. E-mail: raoraowang@hotmail.com.
